# Sulforaphane treatment for autism spectrum disorder: A systematic review

**DOI:** 10.17179/excli2020-2487

**Published:** 2020-06-26

**Authors:** Greer McGuinness, Yeonsoo Kim

**Affiliations:** 1 Central Michigan University, 207 Wightman Hall, 1202 S. Washington Street, Mount Pleasant, MI 48859, U S A

**Keywords:** sulforaphane, Autism Spectrum Disorder, glucosinolates, treatment, CAM

## Abstract

Autism Spectrum Disorder (ASD) is defined as a neurodevelopmental condition characterized by social communication impairment, delayed development, social function deficit, and repetitive behaviors. The Center for Disease Control reports an increase in ASD diagnosis rates every year. This systematic review evaluated the use of sulforaphane (SFN) therapy as a potential treatment option for individuals with ASD. PubMed.gov, PubMed Central, Natural Medicines, BoardVitals, Google Scholar and Medline were searched for studies measuring the effects of SFN on behavior and cognitive function. All five clinical trials included in this systematic review showed a significant positive correlation between SFN use and ASD behavior and cognitive function. The current evidence shows with minimal side effects observed, SFN appears to be a safe and effective treatment option for treating ASD.

## Introduction

Autism Spectrum Disorder (ASD) is defined as a neurodevelopmental condition characterized by social communication impairment, delayed development, social function deficit, and repetitive behaviors (Li et al., 2017[26]; Fung et al., 2016[15]). Defined in 1911 by psychiatrist Eugen Bleuler, who used the term Autism (Greek *autos* to describe extreme social withdrawal) to define a cluster of unique symptoms that were traditionally thought to be described as symptoms of schizophrenia (Landrum and Benham, 2018[25]; Eack et al., 2017[10]). It was not until 1980 (rate of autism was 1:10,000) that the classification of schizophrenia and autism were identified to be two unrelated disorders NAA, n.d.[34]; Miller et al., 2012[31]). Currently the criteria for the classification of ASD are based on impairments in social communication and interactions and restricted, repetitive patterns of behaviors, interests or activities. Common comorbidities such as social anxiety, oppositional defiant disorder, attention-deficit/hyperactivity disorder, and intellectual disability also can be observed in ASD criteria for classification (Masi et al., 2017[30]; Bi et al., 2018[2]).

The Center for Disease Control (CDC) reported currently in the U.S. Autism diagnosis rate is 1:59 (2016). This marks a significant increase from the previous data of 1:68 in 2012 (Center for Disease Control, 2020[7]). Kogan et al. (2018[24]), reported that the most recent statistical rate of Autism for the U.S is now 1:40, which would indicate another significant increase from the previous 2016 CDC statistics. Boys are four times more likely to be diagnosed with Autism compared to girls (1:37 verses 1:51); although identification and early intervention of diagnosing have improved, intellectual functioning, genetics, hormonal factors, environmental and metabolic factors may be the cause and contribute to the ratio difference (Sanchack and Thomas, 2016[48]; Lyall et al., 2017[28]).The most recent spike in diagnosis could have been attributed to the publication of Diagnostic and Statistical Manual of Mental Disorders, 5^th^ ed. (DSM-5), which in 2013 combined four previously separate disorders (Asperger's syndrome, childhoods disintegrative disorder, pervasive developmental disorder, and autism) under the same umbrella diagnosis of ASD (Bölte et al., 2018[4]; Happé et al., 2020[20]). 

Currently to date, there is no U.S. Food and Drug Administration (FDA)-approved treatment for ASD. Pharmacological interventions for ASD are typically targeted to treat specific behaviors such as depression or aggression (psychotropic medication) that significantly impact daily function (Filipek et al., 2006[13]). Many psychotropic medications are used “off-label” and long-term effects for any medication is limited on the developing brain (Filipek et al., 2006[13]; Posey et al., 2008[39]). Many families are worried about potential side effects and are continuously looking for more secure treatment options (Brondino et al., 2015[5]). This has led many to turn towards complementary and alternative medicine (CAM). CAM interventions can be the utilization of dietary supplements, special diets, mind, and body practices and other complementary health approaches. The use of CAM interventions in pediatrics to aid with illnesses and diseases can range up to 76 % (Trudeau et al*.*, 2019[55]). Because autism is individualized, CAM interventions and use are dependent on the symptoms. 

Sulforaphane (SFN: 1-isothiocyanato-4-methylsulfinylbutane), a potent antioxidant, is one CAM intervention that has been studied in ASD and other comorbidities with positive effects (Shirai et al., 2015[51]). SFN is a dietary isothiocyanate that is synthesized from glucosinolate (glucoraphanin), and a precursor found in cruciferous vegetables such as cauliflower, broccoli, kale, cole crops, cabbage, collards and brussels sprouts, mustard, and cress (Guerrero-Beltrán et al., 2012[18]; Carrasco-Pozo et al., 2015[6]). Since its discovery in 1948, this organosulfur compound has been frequently studied and has exhibited multiple biological effects, including antioxidant, antimicrobial, anticancer, anti-inflammatory, anti-aging, neuroprotective, and antidiabetic (Kim and Park, 2016[23]; Fahey and Talalay, 1999[12]; Mokhtari et al., 2017[32]). Several ASD-associated basic physiological pathways that SFN directly impacts are regulation of redox metabolism/oxidative stress, mitochondrial dysfunction, immune dysregulation/neuroinflammation, febrile illness and heat shock response, and synaptic dysfunction (Liu et al., 2016[27]; Ratajczak and Sothern, 2016[41]; Deth et al., 2008[9]).

Over the past six years SFN has been clinically studied and found to have a significant positive impact on individuals with ASD. This systematic review presented findings about the use of SFN as a safe and effective supplement in treating ASD and the impact it had on cognitive and behavioral assessments. While there is still no definitive answer on what exactly causes ASD, the use and research of supplements such as SFN is crucial in providing possible treatment options to alleviate symptoms and behaviors associated with ASD.

## Methods

Research was conducted for this systematic review using the PRISMA guidelines (Prisma, 2015[40]). A literature review was conducted to identify any experimental trials with sulforaphane and participants with a diagnosis of Autism Spectrum Disorder. The databases searched were: PubMed.gov, PubMed Central, Natural Medicines, BoardVitals, Google Scholar and Medline. Keywords used to identify articles; “Sulforaphane,” “Treatment,” “Autism,” “Glucosinolates,” and “CAM”. A total of nine searches were conducted using PubMed.gov, PubMed Central, Natural Medicines, BoardVitals, Google Scholar and Medline using the search terms in a variety of combinations. Articles that were included were from any year, no language limitations, no age limit and not gender specific. Articles were excluded if they were not experimental studies, or the experimental study was used with animals or had other developmental disabilities as a primary diagnosis. Risk of bias was assessed using Cochrane's tool with all-inclusive studies. Primary outcomes to determine if SFN improved ASD function was measured using ASD behavior and cognitive function assessments. 

## Results

**Figure 1:**
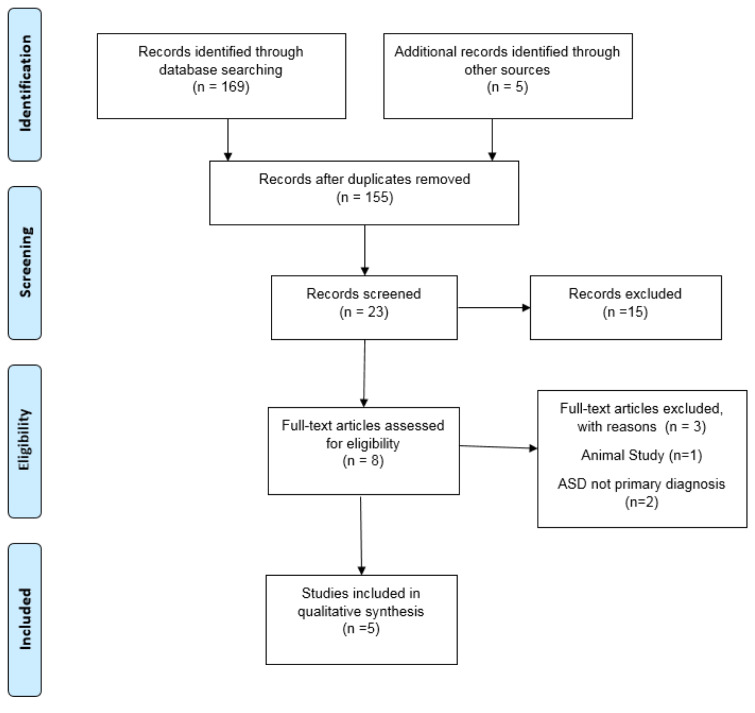
PRISMA Flow Chart. Retrieved from http://www.prisma-statement.org/

**Figure 2:**
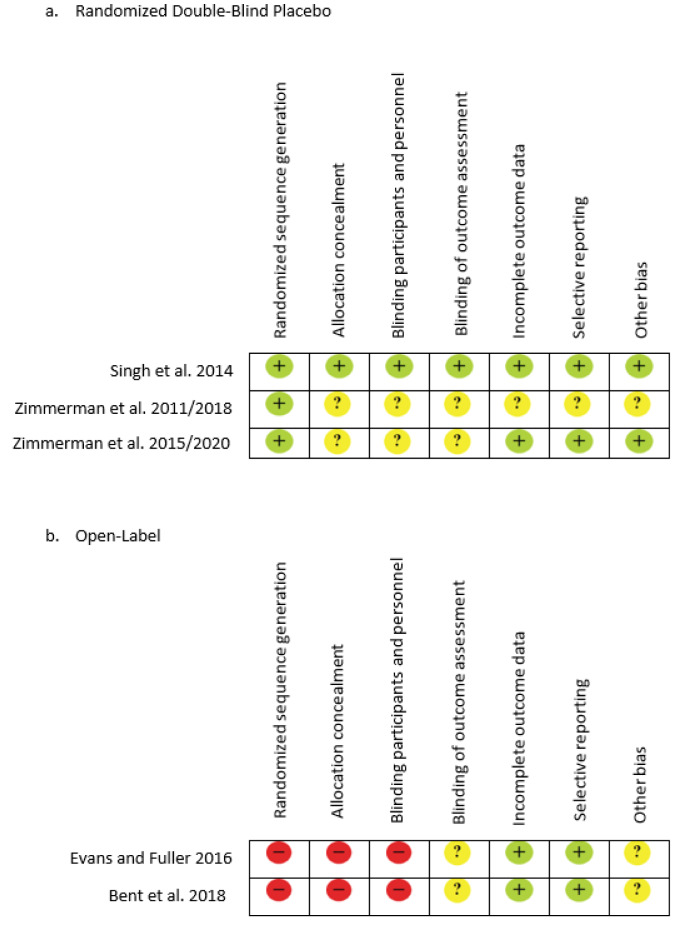
Risk bias assessment of the included studies examining the use of Sulforaphane on individuals with Autism Spectrum Disorder. Symbols: Red indicates high risk, Yellow indicates unclear and Green indicates low risk.

The initial literature search resulted in a total of 169 records from the combination of search terms listed above with an additional five records identified. After screening, 166 were eliminated due to non SFN interventions. A total of eight articles remained after reviewing the studies for content and relevance to the study design. One study was excluded after reading the full text due to the use of animal subjects even though the outcome was based on ASD symptoms, and two others were excluded due to the subjects having schizophrenia as the primary diagnosis, leaving five U.S. studies for the quality synthesis and data extraction. Figure 1(Fig. 1) further explains the process of selecting articles and Table 1(Tab. 1) (References in Table 1: Bent et al., 2018[1]; Evans and Fuller, 2016[11]; Singh et al., 2014[52]; Zimmerman, 2011/2018[61]; Zimmerman, 2015/2020[60]) displays the five articles used for this review by the author, study type, population, supplementation, duration, and study results. Risk of bias was reviewed, the three randomized double-blind articles are low to unclear risk of bias, while the two open-label reviews are unclear to high risk for bias due to study design (Figure 2(Fig. 2); References in Figure 2: Bent et al., 2018[1]; Evans and Fuller, 2016[11]; Singh et al., 2014[52]; Zimmerman, 2011/2018[61]; Zimmerman, 2015/2020[60]). 

### Double-blind randomized control studies

Three double-blind, randomized placebo control trials have been conducted with SFN and ASD. The first original study conducted by Singh et al. (2014[52]), lasted 18 weeks and comprised of 44 males ages 13-27 with a diagnosis of ASD. Twenty-nine subjects were provided with a broccoli sprout extract SFN oral dose based on 50 µmol per 100 lbs of body weight while the control group comprised of fifteen subjects who consumed microcrystalline cellulose placebo. Results found significant improvement in the SFN treatment group versus the placebo. Both Aberrant Behavior Checklist Scores (ABC) and Social Responsiveness Scale Score (SRS) improved with treatment of SFN by 34 % (ABC) and 17 % (SRS), as well as improvement in irritability, lethargy, stereotype, and hyperactivity. Analysis of Clinical Global Impression Improvement Scale (CGI-I) showed improvement with much or very much in social interactions, aberrant behaviors and verbal communication (Singh et al., 2014[52]). Similar findings were observed with two current clinical trials conducted by Zimmerman (2011/2018[61]; 2015/2020[60]; Zimmerman et al., 2018[62]) The first clinical trial (Zimmerman et al., 2018[62]) was a 3 phase, 30-week treatment period with 50 children (boys and girls) age 3-12 years with the diagnosis of ASD. The first 15 weeks of Phase 1 consisted of a 1:1 double-blind placebo trial where 25 children received a placebo, and 25 children received an oral dose of broccoli seed powder SFN 2.2 µmol per kg of body weight. In Phase 2, all children received the same oral dose of broccoli seed powder of SFN (2.2 µmol/kg) from 15-30 weeks and in Phase 3 all children received no treatment for 6 weeks. Preliminary analysis of Ohio Autism Global Impression Scale-Improvement (OACIS-I) showed improvements of 26 % at 7 weeks, 38 % at 15 weeks, 64 % at 22 weeks and 64 % at 30 weeks. Zimmerman's most recent clinical trial (2011/2018[61]) comprised of 44 male adolescents and adults (13-30 years) with autism who were followed for 22 weeks in a 2:1 randomized, double-blind placebo trial. Twenty-nine subjects were randomly selected to receive an oral dose of broccoli sprout extract SFN, 50 µmol per 101lbs of body weight and fifteen subjects consumed an oral dose of microcrystalline cellulose placebo supplement. Although no statistical analysis has been released, raw result scores did show improvement in ABC score, OASCIS-I, and OASCIS-S scores.

### Open-label studies

Following Singh et al. (2014[52]), a follow-up study was conducted by Evans and Fuller (2016[11]). The primary focus of thestudy (2016) was to assess the extent of any notable positive outcome from taking SFN supplementation. This study was comprised of only 6 subjects who were observed for 28 weeks, 10 weeks longer than the original study by Singh et al. (2014[52]). Each participant was given a broccoli sprout extract SFN oral dose based on 50 µmol per 100 lbs of body weight, and assessment scores were based on specific attributes related to ASD symptoms (0=not at all a problem, 1=a problem but slight in degree, 2=moderately serious, and 3=severe in degree). In total 92 attributes were identified as moderately severe or severely affected by their ASD, 74 attributes (80 %) saw some positive changes, and 36 (39 %) saw significant improvements. Participants continued to see improvements passed the longest interval (28 weeks) (Evans and Fuller, 2016[11]). Similar outcomes were observed in Bent et al. (2018[1]) study where 15 children participated in an open-label study who were provided with a broccoli seed and broccoli sprout blend based on 2.5 µmol/lb of body weight. Clinical testing using ABC and SRS scores, as well as urine samples, were used to monitor behaviors pre and post supplementation. After 12 weeks clinical scores showed significant improvements (decreasing score indicates improvements) in ABC score by 7.1 points (17.4 to 3.2) and SRS scores 9.7 points (18.7 to 0.8).

Evans' and Fuller's study (2016[11]) was the first to show clinical improvements with an assessment of urinary metabolite levels. Some of the correlated improved marks were in oxidative stress, redox metabolism, amino acid metabolism, neurotransmitter-related metabolites, hormones, and chemical sphingomyelin (Bent et al., 2018[1]).

The most common side effects reported in all three double blind studies (range 12-19 %) of participants were insomnia, flatulence, constipation, weight gain, vomiting, diarrhea, increased aggression, and exacerbation of seasonal allergies (Singh et al., 2014[52]; Zimmerman 2011/2018[61]; 2015/2020[60]; Zimmerman et al., 2018[62]). Two participants in the Singh et al. study (2014[52]) had a single unprovoked seizure, one was three weeks after starting SFN, however the participant had an undisclosed history of recent seizure activity; the second had an unprovoked seizure three weeks after SFN trial completed and a history of seizures (> 1 year) which were well-controlled with antiepileptic medication. Only six families reported new adverse events during Bent et al. (2018[1]) open label trial after starting SNF supplementation. Families reported: one nausea and vomiting, one stomach flu, one inflammation in the esophagus, one weight gain, one weight loss, and one ruptured appendix with no long-term complications. No side effects were reported by the participants in Evans' and Fuller's trial (2016[11]).

## Discussion

This systematic review included five experimental studies; three randomized double-blind placebo and two open-label studies of the effects SFN has on ASD. After reviewing and comparing clinical data, the results have shown a significant improvement with behavior, social and cognitive scores with SFN use. The clinical outcomes from the first trial by Singh et al. (2014[52]) even prompted a follow- up case report (Lynch et al., 2017[29]). Nine individuals from original SFN trial group had continued to use SFN even after the clinical trial was over (3 years post) and noted seeing continual positive effects on ASD symptoms. The clinical use of SFN in ASD and other neurological disorders is growing, and more clinical trials are currently being conducted in hopes to strengthen its use as a positive treatment option NCT02677051 (Johnson, 2016/2019[22]), NCT02909959 (Politte, 2016/2020[38]), NCT02654743 (Hendren, 2016/2019[21]), and NCT02879110 (Ou, 2016/2019[36]).

Sulforaphane is a natural phytochemical; dormant and or germinating seeds contain the highest concentration of glucosinolates followed by developing inflorescences, siliques (fruits), young leaves, roots, and mature leaves (Yang et al., 2016[57]; Yagishita et al., 2019[56]). It is converted from glucoraphanin by myrosinase, β-thioglucoside glucohydrolase, during the damage of plant integrity or by hydrolysis by uncharacterized β-thioglucosidases of the gut microflora (Sedlak et al., 2017[49]; Yagishita et al., 2019[56]). Its molecular weight of 177g/mol and its lipophilicity ability allow it to passively diffuse into the enterocytes and become rapidly absorbed by the body (NCBI, 2020[35]; Tarozzi et al., 2013[54]). It can also pass through the blood-brain-barrier and accumulate in the central nervous system providing a neuroprotective effect (Zhang, 2017[58]; Sivapalan et al., 2018[53]). Several research studies have shown SFN can quickly be absorbed with some results showing a peak concentration within 1-3 hrs and disappearance within 12-24 hours (Zhang, 2017[58]). Individuals with ASD tend to have lower levels of sulfur-containing compounds which are essential to the human body to conduct key regulations, as well as have abnormal levels of specific biomarkers such as: oxidative stress, glutathione, and mitochondrial dysfunction (Bittker, 2016[3]; Rossignol and Frye, 2011[45], 2014[46]).

The use of clinical laboratory biomarkers has given many answers to the possible reasons for the social and cognitive impairments in ASD. Even though ASD is defined as a behavior disorder, many individuals with ASD tend to share similarities when it comes to biochemistry (Frustaci et al., 2012[14]). Oxidative stress is affected by the balance, or lack thereof, between pro-oxidants and antioxidant systems in the cells (Ghanizadeh et al., 2012[17]). The damage to cellular tissue caused by free radicals like reactive oxygen species (ROS) is what leads to oxidative stress and or mitochondrial dysfunction (Rossignol and Frye, 2011[45]). This damage ultimately contributes to the progress and clinical diagnosis of autism (Chauhan and Chauhan, 2006[8]). Mitochondrial dysfunction may be a direct result from elevated levels of OS, since ROS is generated from pro-oxidant environmental toxicants and activated immune cells (Rose et al., 2015[42]). 

To combat oxidative stress, the body has a primary antioxidant that is responsible for the intracellular defense of redox reduction/oxidation process (RO) called glutathione. Glutathione is imperative for normal cell function, viability and aids in the excretion of metals, which has clinically shown to be higher in ASD individuals (Ghanizadeh et al., 2012[17]; Rose et al., 2012[43]). It may also be the reason why the autism rate in males is higher than females since males have lower glutathione levels, which puts them at a more vulnerable state for oxidative stress to occur (Rossignol and Bradstreet, 2008[44]). 

SFN is not a direct-acting antioxidant or pro-oxidant; however, *in vivo* studies have shown that it indirectly increases antioxidant capacity and its ability to cope with oxidative stress (Fahey and Talalay, 1999[12]). Several animal studies (Zhang and Talalay, 1994[59]; Morroni et al., 2013[33]; Pearson et al., 2016[37]) have shown SFN to directly raise GSH levels by stimulating the Nrf2-Antioxidant Response Elements (ARE) and increasing the cellular antioxidant defense. It also protects against toxicity in DAergic cells, induces antioxidant enzymes, reduces tissue/cell damage, prevents hepatic damage, and ultimately aids in the preservation of mitochondrial function and decreases oxidative stress (Guerrero-Beltrán et al., 2012[18]; Han et al., 2007[19]; Gaona-Gaona et al., 2011[16]). Other neurological and psychiatric disorders such as Alzheimer's disease, Parkinson's Disease, schizophrenia, and bipolar disorder have similar associated abnormal levels of oxidative biomarkers (Salim, 2017[47]). Schizophrenia has long been separated from the classification of ASD. However, in a pilot study and an open label study, SFN showed positive improvements in cognitive function as well as specific biomarker levels similar to ASD (Sedlak et al., 2017[49]; Shiina et al., 2015[50]). These results further strengthen the use of SFN on neurodevelopment disorders and improvement in specific biomarkers.

Several strengths were found in the current systematic review. First, the use of randomized double-blind placebo testing in three of the reviews (Singh et al., 2014[52]; Zimmerman, 2011/2018[61], 2015/2020[60]; Zimmerman et a., 2018[62]) provides insight for further research and allows the ability to track data overtime. The use of randomized double-blind testing is a gold standard method of research to compare data with a control group with the intervention being presented. Second, in four of the studies (Singh et al., 2014[52]; Zimmerman, 2011/2018[61], 2015/2020[60]; Zimmerman et a., 2018[62]; Bent et al., 2018[1]) standardized screening assessments were used prior and post supplement use. Third, using specialist ASD screening assessments to monitor any improvements in multiple areas may provide future research and studies with beneficial information. 

The main limitation to the findings is that most had a short duration period of 12-30 weeks and contained small sample size 6-30 participants. It would be beneficial to have a longer duration clinical trial to see if improvements continue, accelerate or decline after a specific time frame. Increasing sample size maybe difficult due to the characteristic of the subjects. Children with ASD may not take the supplement or may have a difficult time with follow up clinical appointments. Evans' and Fuller's study (2016[11]) was the only experiment that did not use a standardized screening assessment pre and post SFN supplement making replication difficult. 

With the rapidly increasing rate of ASD occurring in the U.S. and with no definitive cause or pharmaceutical treatment options, families are looking elsewhere to help improve their loved one's quality of life. Using CAM options such as SFN is giving hope that treatment options are a possible intervention. The current available evidence shows with minimal side effects observed, SFN appears to be a safe and effective treatment option for treating ASD and other neurological disorders.

## Conflict of interest

The authors declare that there is no conflict of interest.

## Funding

This research received no specific grant from any funding agency.

## Figures and Tables

**Table 1:**
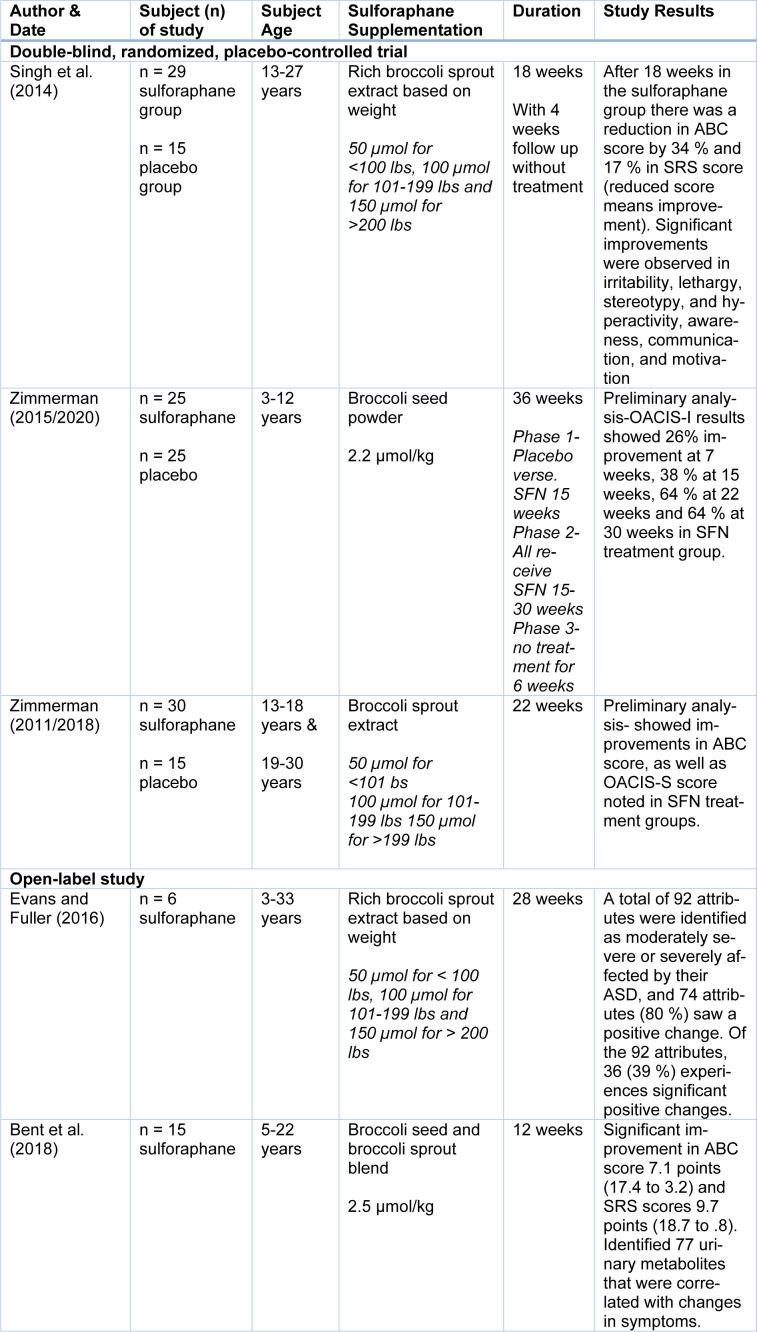
Results of experimental studies conducted with Sulforaphane and Autism Spectrum Disorder
